# BE GENERATIVE TO ACCEPT DEATH: ROLE OF MEANING IN LIFE MODERATED BY AGE AND PERCEIVED IMPACT

**DOI:** 10.1093/geroni/igz038.2890

**Published:** 2019-11-08

**Authors:** Nicole Long Ki Fung, Steven Tsun-wai Chu, Helene Hoi-lam Fung

**Affiliations:** 1 the Chinese University of Hong Kong, Hong Kong, Hong Kong; 2 The Chinese University of Hong Kong, Hong Kong, Not Applicable, Hong Kong

## Abstract

Meaning-management theory (MMT) suggests living a meaningful life leads to higher death acceptance. This paper investigates how generativity, i.e. the intention to bring benefits to the next generation, can affect death acceptance through achieving meaning in life (MIL). 343 participants in Hong Kong (aged 18-90) filled in a questionnaire as part of “Age(ing) as Future” project. Generativity positively correlates with death acceptance. MIL fully mediates the effect of generativity on death acceptance (r(343)=.132, p=.014). The effect of generativity on MIL might differ by age and perceived influences of generative acts. As speculated, the mediation is moderated by Age and Transformational Future Time Perspective (TFTP; generative impact that expands future time perspective). In older adults with lower TFTP, generativity no longer predicts MIL and the mediation was nonsignificant. The results provide empirical support to MMT and emphasise the importance of perceived impact and the needs in different developmental stage.

